# Supplementary material for: Evolutionary characteristics of intergenic transcribed regions indicate rare novel genes and widespread noisy transcription in the Poaceae
Source: Sci Rep. 2019 Aug 20;9:12122. doi: 10.1038/s41598-019-47797-y (PMC6702216; doi:10.1038/s41598-019-47797-y)
Supplement: Supplementary file 1 — Supplemental Information [file 41598_2019_47797_MOESM1_ESM.pdf]

Supplemental Information

Evolutionary characteristics of intergenic transcribed regions indicate rare novel genes and widespread noisy transcription in the Poaceae

John P. Lloyd, Megan J. Bowman, Christina B. Azodi, Rosalie P. Sowers, Gaurav D. Moghe, Kevin L. Childs, Shin-Han Shiu

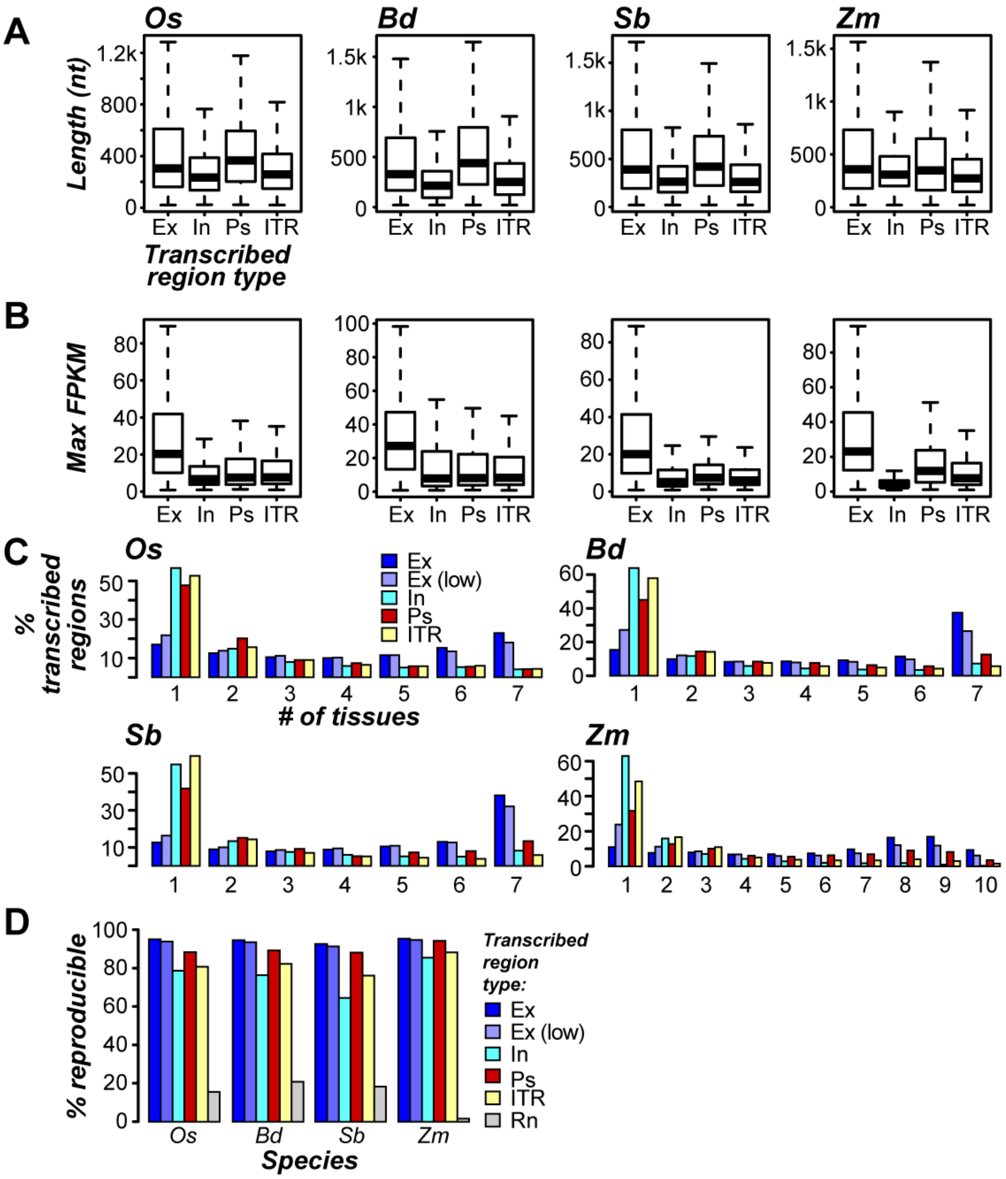

Fig. S1. Expression characteristics of transcribed regions in four Poaceae species. **(A)** Boxplots of length distributions among transcribed regions that overlap exon (Ex), intron (In), pseudogene (Ps), or intergenic (ITR) regions. Nt: nucleotides. Species abbreviation follows that of **Fig. 1**. **(B)** Boxplots of maximum FPKM distributions among all tissues. **(C)** Histograms of expression breadth (# of tissues with expression evidence) for transcribed regions. Ex (low): a subset of exons with expression levels ±5% of intergenic transcribed regions. **(D)** Percentage of transcribed regions that are reproducible across biological replicate leaf transcriptome datasets. Intergenic sequences were randomly-sampled to determine the background expected reproducibility (Rn).

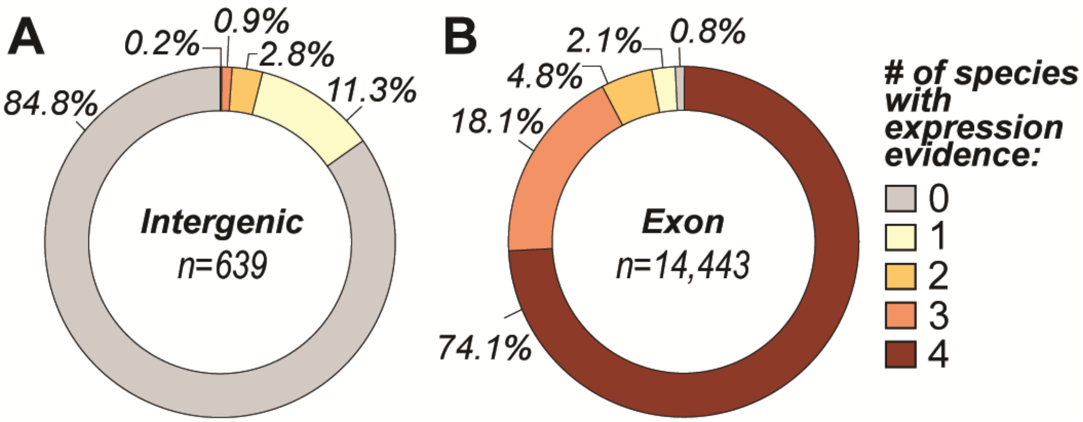

Fig. S2. Ring plots displaying the number of species with evidence of expression for sequence blocks conserved across all four species. Sequence blocks shown were composed of only intergenic (**A**) or only exon (**B**) sequences from all species.

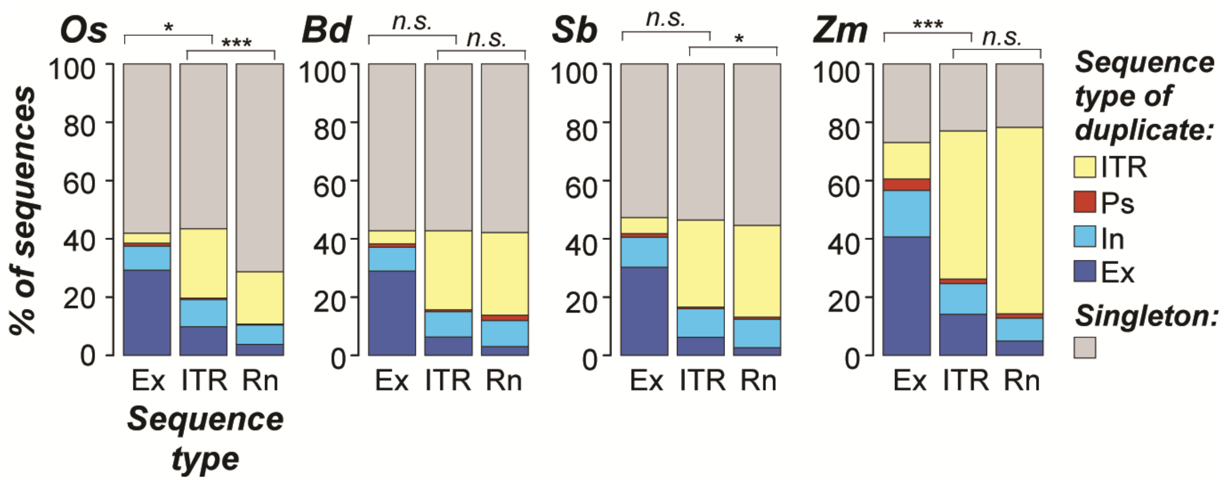

Fig. S3. Distributions of duplication types among transcribed exons (Ex, x-axis), ITRs, and random intergenic (Rn) sequences. Fisher's exact tests were used to test significance between the proportion of sequences that were duplicated (sum of Ex, In, Ps, and ITR duplicate proportions) versus proportion of singletons. Although duplication rates between transcribed exons and ITRs could be significantly different, almost all differences were less than 5%, indicating that the presence of a duplicate is not informative to whether a sequence is likely part of an annotated gene. *: $p<0.05$, ***: $p<0.001$, n.s.: not significant, $p\geq0.05$, In: intron, Ps: pseudogene.

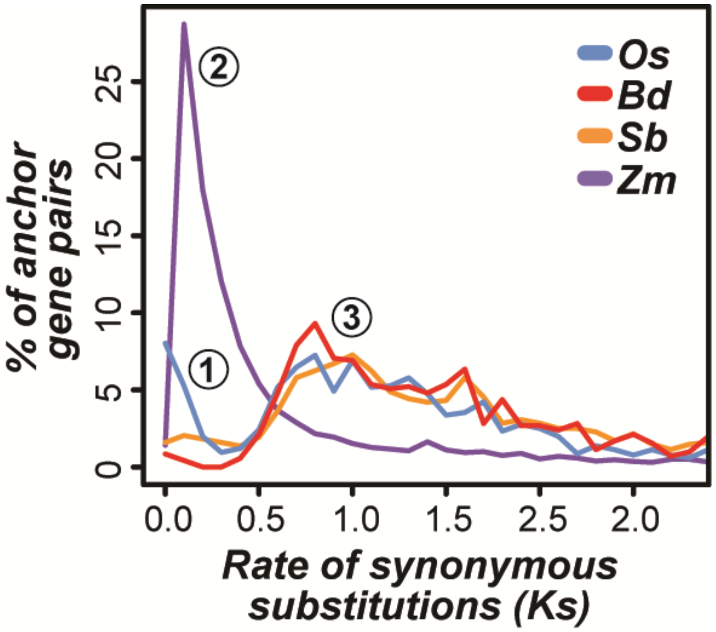

Fig. S4. Distributions of synonymous substitution rates (*Ks*) between anchor genes of within-species collinear gene blocks. Circled numbers indicate *Ks* peaks associated with (1) a low-*Ks* large-scale duplication in rice, (2) a recent whole genome duplication (WGD) in maize, and (3) the ρ and σ WGD events. Average *Ks* values among ρ and σ duplicates have been estimated at 0.9 and 1.7, respectively [31,32]. *Ks* distributions for these two events are highly overlapping and cannot be effectively distinguished. Due to uncertain origin and timing of the low-*Ks* rice duplication (1) [51], duplicates from this event were not included in further analysis. *Os*: rice, *Bd*: *B. distachyon*, *Sb*: sorghum; *Zm*: maize.

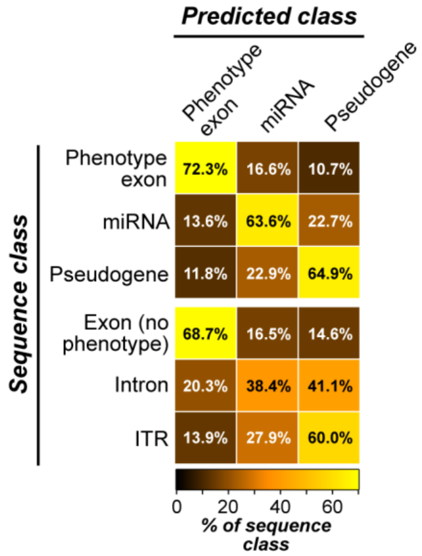

Fig. S5. Heatmap of three-class prediction model performance. Sequences among six sequence classes (rows) were predicted as phenotype exon-like, transcribed miRNA-like, or transcribed pseudogene-like (columns) based on a three-class random forest prediction model. Heatmap colors and numbers correspond to the percentages of a given sequence class predicted as a given class.

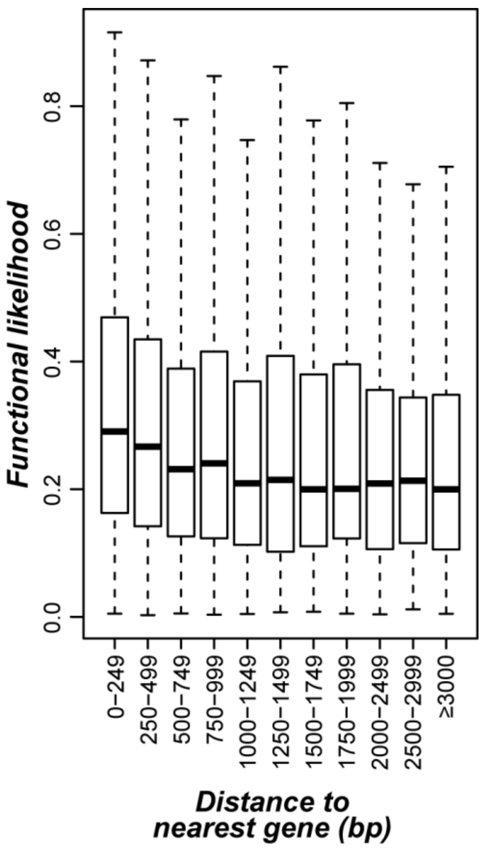

Fig. S6. Boxplots of the relationship between distance to nearest gene and predicted functional likelihood for ITRs.

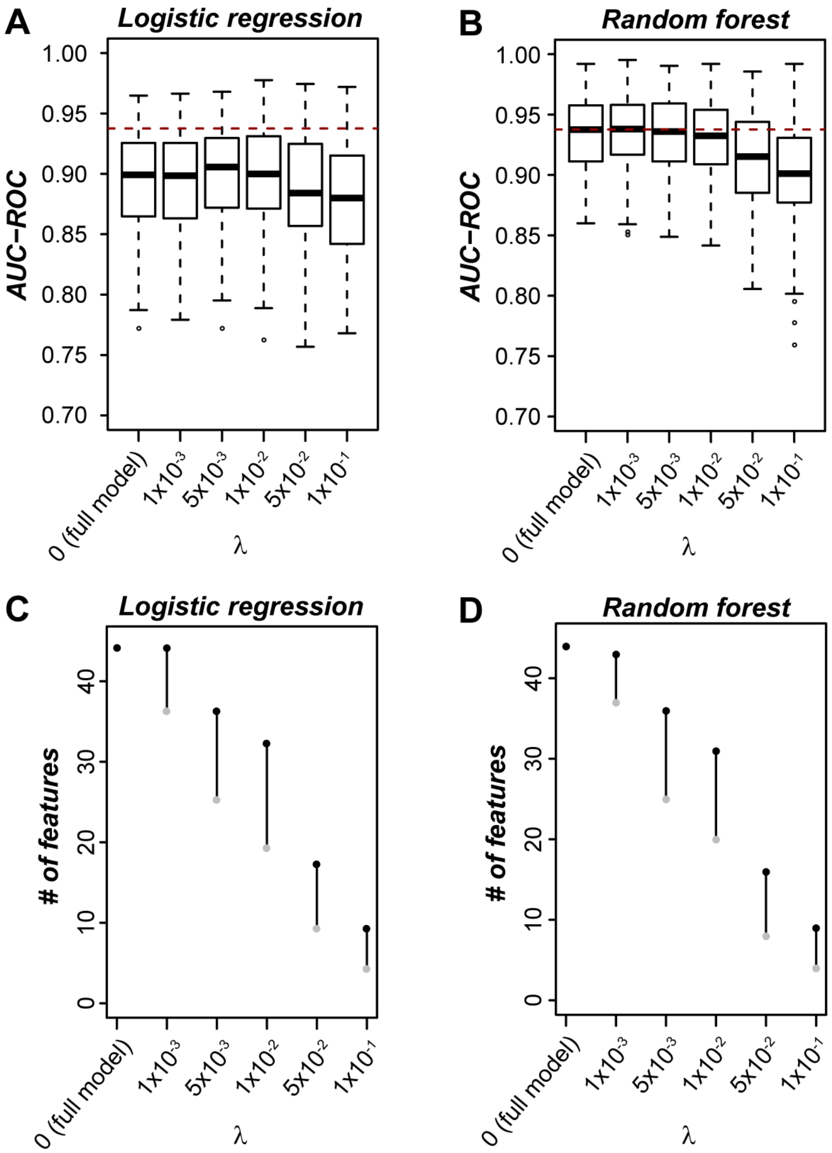

Fig. S7. Boxplots of AUC-ROC values for functional prediction models generated using logistic regression (A) or random forest (B) based on 100 bootstrap replicates of models trained on balanced sets of 450 randomly-selected phenotype exons and expressed pseudogenes and applied to independent balanced sets of 50 randomly-selected phenotype exons and expressed pseudogenes. Dashed red lines in (A, B) indicate median AUC-ROC of the full random forest model (λ=0). AUC-ROC distributions are also shown for models developed following feature selection by least absolute shrinkage and selection operator (LASSO), where a higher λ parameter selects fewer features (C, D). Ranges of feature counts used to build logistic regression (C) and random forest (D) models are shown. Gray and black circles indicate the lowest and highest feature count, respectively, from 100 bootstrap replicates.

Table S1. NCBI-SRA datasets used in transcribed region identification.

Species	SRA ID	Tissue	Reads (in millions)
B. distachyon	SRR349785	Leaves - 20 days	24.1
B. distachyon	SRR349786	Early inflorescence	17.6
B. distachyon	SRR349787	Emerging inflorescence	23.1
B. distachyon	SRR352137	Pistil	17.7
B. distachyon	SRR352138	Embryo - 25 DAP	22.4
B. distachyon	SRR352139	Seed - 5 DAP	25.9
B. distachyon	SRR352140	Anther	26.1
B. distachyon	SRR352141	Seed - 10 DAP	25.8
B. distachyon	SRR352142	Endosperm - 25 DAP	27.4
B. distachyon	SRR352143	Leaves - 20 days	32.5
B. distachyon	SRR352144	Embryo - 25 DAP	26.3
Maize	SRR189760	Pre-emergence cob	18.3
Maize	SRR189761	Leaves - 20 days	17.1
Maize	SRR189762	Pre-pollination tassel	19.8
Maize	SRR189763	Post-emergence cob	18.3
Maize	SRR189768	Endosperm - 25 DAP	23.6
Maize	SRR189769	Anther	27
Maize	SRR189770	Ovule	29.9
Maize	SRR189771	Pollen	27.9
Maize	SRR189772	Mature silk	18.9
Maize	SRR189773	Seed - 10 DAP	26.4
Maize	SRR189774	Leaves - 20 days	32.7
Maize	SRR393016	Post-pollination tassel	18.6
Maize	SRR393017	Seed - 5 DAP	19.2
Maize	SRR393018	Embryo - 25 DAP	19.9
Rice	SRR352184	Leaves - 20 days	24.3
Rice	SRR352187	Emerging inflorescence	23.8
Rice	SRR352189	Early inflorescence	29.4
Rice	SRR352190	Anther	28.3
Rice	SRR352192	Pistil	17.2
Rice	SRR352194	Seed - 5 DAP	16.4
Rice	SRR352204	Embryo - 25 DAP	21.4
Rice	SRR352206	Endosperm - 25 DAP	24.4
Rice	SRR352207	Seed - 10 DAP	24.9
Rice	SRR352209	Endosperm - 25 DAP	24.2
Rice	SRR352211	Leaves - 20 days	31.8
Sorghum	SRR349643	Leaves - 20 days	24.1
Sorghum	SRR349644	Emerging inflorescence	27.1
Sorghum	SRR349645	Seed - 5 DAP	24.4
Sorghum	SRR349646	Early inflorescence	30.6
Sorghum	SRR349754	Pistil	29.2
Sorghum	SRR349767	Embryo - 25 DAP	24.7
Sorghum	SRR349768	Endosperm - 25 DAP	20.5
Sorghum	SRR349769	Anther	22.9
Sorghum	SRR349771	Seed - 10 DAP	26.1
Sorghum	SRR349772	Leaves - 20 days	33.1

Table S2. Machine learning feature list and single-feature AUC-ROC performance.

Category	Feature	AUC-ROC
DNA Methylation	CG methylation level - embryo	0.56
DNA Methylation	CG methylation level - endosperm	0.55
DNA Methylation	CG methylation level - leaf	0.51
DNA Methylation	CG methylation level - panicle	0.54
DNA Methylation	CHG methylation level - embryo	0.57
DNA Methylation	CHG methylation level - endosperm	0.57
DNA Methylation	CHG methylation level - leaf	0.65
DNA Methylation	CHG methylation level - panicle	0.58
DNA Methylation	CHH methylation level - embryo	0.57
DNA Methylation	CHH methylation level - endosperm	0.54
DNA Methylation	CHH methylation level - leaf	0.62
DNA Methylation	CHH methylation level - panicle	0.55
DNA Methylation	Gene body methylation - embryo	0.64
DNA Methylation	Gene body methylation - endosperm	0.58
DNA Methylation	Gene body methylation - leaf	0.64
DNA Methylation	Gene body methylation - panicle	0.65
Histone marks	# of activation-related marks	0.63
Histone marks	# of repression-related marks	0.62
Histone marks	H3K23ac - coverage	0.51
Histone marks	H3K27me3 - coverage	0.56
Histone marks	H3K36me1 - coverage	0.52
Histone marks	H3K36me2 - coverage	0.62
Histone marks	H3K36me3 - coverage	0.67
Histone marks	H3K4me2 - coverage	0.73
Histone marks	H3K4me3 - coverage	0.54
Histone marks	H3K9ac - coverage	0.51
Histone marks	H4K12ac - coverage	0.51
Histone marks	H4K16ac - coverage	0.60
Nucleosome occupancy	Nucleosome occupancy - ave.	0.67
Sequence conservation	Conserved nucleotide block - coverage	0.72
Sequence conservation	Cross-species sequence similarity - max. E-value	0.79
Sequence conservation	phastCons score - median	0.72
Transcription activity	Breadth	0.72
Transcription activity	Level - anther	0.53
Transcription activity	Level - early inflorescence	0.80
Transcription activity	Level - embryo	0.69
Transcription activity	Level - emerging inflorescence	0.78
Transcription activity	Level - endosperm	0.60
Transcription activity	Level - leaves	0.66
Transcription activity	Level - max.	0.78
Transcription activity	Level - median	0.75
Transcription activity	Level - pistil	0.67
Transcription activity	Level - seed (10 DAP)	0.62
Transcription activity	Level - seed (5 DAP)	0.64

Table S3. NCBI-SRA datasets used in gene annotation.

Species	SRA Number(s)	Bases (Mbp)	Read Length (bp)	Paired or Single
B. distachyon	SRR921329,SRR921327	942.3	35	Single
B. distachyon	SRR921328,SRR921326	1020.3	35	Single
B. distachyon	SRR921318	612.6	49	Single
B. distachyon	SRR921317	616.8	49	Single
B. distachyon	SRR352144	919.9	35	Single
B. distachyon	SRR352142	957.8	35	Single
B. distachyon	SRR352141	901.8	35	Single
B. distachyon	SRR352140	912.1	35	Single
B. distachyon	SRR352139	907.3	35	Single
B. distachyon	SRR352138	784	35	Single
B. distachyon	SRR349787	809.8	35	Single
B. distachyon	SRR349786	616	35	Single
B. distachyon	SRR349785	965.1	40	Single
B. distachyon	SRR035253	1400	36	Single
B. distachyon	SRR035254	1900	36	Single
B. distachyon	SRR035255	376.8	36	Single
B. distachyon	SRR035256	1600	36	Single
B. distachyon	SRR035257	3400	36	Single
B. distachyon	SRR035258	2300	36	Single
Sorghum	SRR299250,SRR299249,SRR299248	5300	51, 76	Single
Sorghum	SRR299247,SRR299246,SRR299245	4500	51	Single
Sorghum	SRR299244,SRR299243,SRR299242	4500	51	Single
Sorghum	SRR299241,SRR299240,SRR299239	4100	51	Single
Sorghum	SRR299238,SRR299237,SRR299236	4700	51	Single
Sorghum	SRR299235,SRR299234,SRR299233	4600	51	Single
Sorghum	SRR299232,SRR299231,SRR299230	4200	51	Single
Sorghum	SRR299229,SRR299228,SRR299227	4300	51	Single
Sorghum	SRR349772	1200	35	Single
Sorghum	SRR349771	914.1	35	Single
Sorghum	SRR349769	800.8	35	Single
Sorghum	SRR349768	719	35	Single
Sorghum	SRR349767	865.5	35	Single
Sorghum	SRR349754	1000	35	Single
Sorghum	SRR349646	1100	35	Single
Sorghum	SRR349645	974.8	40	Single
Sorghum	SRR349644	1100	40	Single
Sorghum	SRR349643	963.8	40	Single
Sorghum	DRR001053	3000	76	Single
Sorghum	DRR001055,DRR001054	2500	76	Single
Rice	DRR000766,DRR000755	1140	51	Single
Rice	DRR000760	549.5	51	Single
Rice	DRR000759	499.3	51	Single
Rice	DRR000767,DRR000756	1038.9	51	Single
Rice	DRR000758,DRR000764	700.4	51	Single
Rice	DRR000746,DRR000748	1355.9	51	Single
Rice	DRR000752	993.1	51	Single
Rice	DRR000751	975.3	51	Single
Rice	DRR000763,DRR000753,DRR000749	2695.3	51	Single
Rice	DRR000761	508.2	51	Single
Rice	DRR000762,DRR000769	1029.5	51	Single
Rice	DRR000745	941.3	51	Single

Rice	DRR000754,DRR000765	1167	51	Single
Rice	DRR001024,DRR001031,DRR001038	1677.1	51	Single
Rice	DRR001048	1600	76	Single
Rice	DRR001040,DRR001026,DRR001033	1941.3	51	Single
Rice	DRR001045	1600	76	Single
Rice	DRR001046	1600	76	Single
Rice	DRR001050	1600	76	Single
Rice	DRR001027,DRR001034,DRR001041	1725.7	51	Single
Rice	DRR001051	1600	76	Single
Rice	DRR001039,DRR001025,DRR001032	1627.4	51	Single
Rice	DRR001047	1600	76	Single
Rice	DRR001049	1600	76	Single
Rice	DRR001029,DRR001036,DRR001043	2084.5	51	Single
Rice	SRR447126	781.1	71	Paired
Rice	SRR447125	552.8	75	Paired
Rice	SRR447124	817.6	96	Paired
Rice	SRR447123	1300	96	Paired
Rice	SRR447122	1300	71	Paired
Rice	SRR447121	1400	96	Paired
Rice	SRR447120	517.3	75	Paired
Rice	SRR447119	764.6	75	Paired
Rice	SRR447118	904.1	75	Paired
Rice	SRR447117	1400	96	Paired
Rice	SRR408749,SRR408748	538.2	76	Single
Rice	SRR408747,SRR408746,SRR408745	887.7	76	Single
Rice	SRR408744,SRR408743,SRR408742	935.9	76	Single
Rice	SRR408741,SRR408740,SRR408739	1166.7	76	Single
Rice	SRR408738,SRR408737	653.6	76	Single
Rice	SRR408736,SRR408735	792.8	76	Single
Rice	SRR408734,SRR408733,SRR408732	1348.1	76	Single
Rice	SRR408731,SRR408730,SRR408729	940.2	76	Single
Rice	SRR976343,SRR976342	9000	100	Single
Rice	SRR976337,SRR976336	6400	100	Single
Rice	SRR976339,SRR976340	6700	100	Single
Rice	SRR1117110	6200	90	Paired
Rice	SRR1117184	6200	90	Paired
Rice	SRR1106688	3500	90	Paired
Rice	SRR1106687	3500	90	Paired

Table S4. Parameters tested for syntenic block identification.

Species	Block type	Minimum gene pairs (#)	Maximum gene gaps (#)	Anchor genes (#)	% of genes that anchor blocks	Megabases covered by blocks	% of genome covered by blocks
Rice	Within-species	10	10	2660	6.9	57.5	15.4
		10	15	3990	10.3	110.6	29.6
	Between-species	5	0	156	0.4	0.8	0.2
		10	1	1466	3.8	12.5	3.3
		10	2	4943	12.8	48.0	12.9
		20	2	1381	3.6	14.0	3.7
		10	3	8311	21.5	92.3	24.7
		10	5	11906	30.8	154.9	41.5
B. distachyon	Within-species	10	10	1764	5.5	42.2	15.6
		10	15	2763	8.7	79.2	29.2
	Between-species	5	0	156	0.5	0.8	0.3
		10	1	1466	4.6	11.1	4.1
		10	2	4946	15.5	44.0	16.2
		20	2	1381	4.3	12.1	4.5
		10	3	8311	26.1	83.4	30.7
		10	5	11785	37.0	137.4	50.7
Sorghum	Within-species	10	10	2121	6.0	62.3	9.5
		10	15	3434	9.6	180.8	27.5
	Between-species	5	0	184	0.5	1.3	0.2
		10	1	749	2.1	12.5	3.3
		10	2	3599	10.1	43.0	6.5
		20	2	891	2.5	10.1	1.5
		10	3	6927	19.5	97.8	14.9
		10	5	11419	32.1	198.2	30.1
Maize	Within-species	10	10	7263	17.9	762.8	37.0
		10	15	9160	22.6	1121.3	54.4
	Between-species	5	0	184	0.5	5.2	0.3
		10	1	749	1.8	7.0	0.3
		10	2	3704	9.1	156.0	7.6
		20	2	891	2.2	36.2	1.8
		10	3	7418	18.3	380.3	18.5
		10	5	13240	32.6	817.2	39.7

Table S5. NCBI-SRA datasets used in histone mark peak identification.

Histone mark	SRA run id	Total protein or H3 control available?	Reads (M)	Activation associated?
H3K23ac	SRR2046532	No	25.1	Yes
H3K27me3	SRR034648	No	3.3	No
H3K27me3	SRR034649	No	4.6	No
H3K27me3	SRR097872	Yes	5.1	No
H3K27me3	SRR097873	Yes	5.9	No
H3K27me3	SRR570427	No	12	No
H3K36me1	SRR2467245	Yes	13	Yes
H3K36me2	SRR2467246	Yes	14.5	Yes
H3K36me3	SRR094791	No	14.4	Yes
H3K36me3	SRR2467247	Yes	13.6	Yes
H3K4me2	SRR094790	No	20.9	Yes
H3K4me3	SRR034622	No	4.5	Yes
H3K4me3	SRR034623	No	2.9	Yes
H3K9ac	SRR034635	No	3.9	Yes
H3K9ac	SRR034636	No	4.3	Yes
H3K9ac	SRR034637	No	5.8	Yes
H4K12ac	SRR094792	No	20.8	Yes
H4K16ac	SRR2046533	No	25.2	No

Table S6. Parameters tested for DNA methylation level feature calculation.

Cytosine context	Minimum reads	Minimum C sites	AUC-ROC
CHG	1	1	0.624
CHG	2	1	0.624
CHG	2	2	0.624
CHG	3	1	0.626
CHG	3	2	0.625
CHG	3	3	0.623
CHG	4	1	0.625
CHG	4	2	0.624
CHG	4	3	0.623
CHG	4	4	0.620
CHG	5	1	0.625
CHG	5	2	0.624
CHG	5	3	0.622
CHG	5	4	0.620
CHG	5	5	0.621
CHG	6	1	0.625
CHG	6	2	0.624
CHG	6	3	0.622
CHG	6	4	0.620
CHG	6	5	0.621
CHG	6	6	0.622
CHG	7	1	0.624
CHG	7	2	0.623
CHG	7	3	0.622
CHG	7	4	0.620
CHG	7	5	0.621
CHG	7	6	0.622
CHG	7	7	0.624
CHG	8	1	0.624
CHG	8	2	0.623
CHG	8	3	0.622
CHG	8	4	0.620
CHG	8	5	0.621
CHG	8	6	0.622
CHG	8	7	0.624
CHG	8	8	0.621
CHG	9	1	0.624
CHG	9	2	0.623
CHG	9	3	0.622
CHG	9	4	0.620
CHG	9	5	0.621
CHG	9	6	0.622
CHG	9	7	0.624
CHG	9	8	0.621
CHG	9	9	0.618
CHG	10	1	0.623
CHG	10	2	0.623
CHG	10	3	0.621
CHG	10	4	0.619
CHG	10	5	0.620
CHG	10	6	0.621
CHG	10	7	0.623
CHG	10	8	0.621
CHG	10	9	0.617
CHG	10	10	0.615
CHG	11	1	0.623
CHG	11	2	0.622
CHG	11	3	0.621
CHG	11	4	0.619
CHG	11	5	0.620
CHG	11	6	0.621
CHG	11	7	0.623
CHG	11	8	0.621
CHG	11	9	0.617
CHG	11	10	0.615
CHG	12	1	0.623
CHG	12	2	0.623
CHG	12	3	0.622
CHG	12	4	0.620
CHG	12	5	0.620
CHG	12	6	0.621
CHG	12	7	0.623
CHG	12	8	0.620
CHG	12	9	0.617
CHG	12	10	0.615
CHG	13	1	0.623
CHG	13	2	0.623
CHG	13	3	0.622
CHG	13	4	0.620
CHG	13	5	0.620
CHG	13	6	0.621
CHG	13	7	0.623
CHG	13	8	0.620
CHG	13	9	0.617
CHG	13	10	0.615
CHG	14	1	0.622
CHG	14	2	0.622
CHG	14	3	0.621
CHG	14	4	0.619
CHG	14	5	0.619
CHG	14	6	0.620
CHG	14	7	0.622
CHG	14	8	0.620
CHG	14	9	0.617
CHG	14	10	0.615
CHG	15	1	0.622
CHG	15	2	0.622
CHG	15	3	0.621
CHG	15	4	0.619
CHG	15	5	0.620
CHG	15	6	0.621
CHG	15	7	0.623
CHG	15	8	0.621
CHG	15	9	0.617
CHG	15	10	0.615
CHG	16	1	0.622
CHG	16	2	0.622
CHG	16	3	0.621
CHG	16	4	0.619
CHG	16	5	0.620
CHG	16	6	0.621
CHG	16	7	0.623
CHG	16	8	0.621
CHG	16	9	0.617
CHG	16	10	0.615
CHG	17	1	0.624
CHG	17	2	0.623
CHG	17	3	0.623
CHG	17	4	0.621
CHG	17	5	0.622
CHG	17	6	0.623
CHG	17	7	0.623

CHG	17	8	0.621
CHG	17	9	0.617
CHG	17	10	0.615
CHG	18	1	0.623
CHG	18	2	0.623
CHG	18	3	0.622
CHG	18	4	0.621
CHG	18	5	0.622
CHG	18	6	0.623
CHG	18	7	0.623
CHG	18	8	0.620
CHG	18	9	0.617
CHG	18	10	0.615
CHG	19	1	0.623
CHG	19	2	0.623
CHG	19	3	0.622
CHG	19	4	0.621
CHG	19	5	0.623
CHG	19	6	0.623
CHG	19	7	0.623
CHG	19	8	0.620
CHG	19	9	0.617
CHG	19	10	0.615
CHG	20	1	0.624
CHG	20	2	0.624
CHG	20	3	0.623
CHG	20	4	0.623
CHG	20	5	0.624
CHG	20	6	0.624
CHG	20	7	0.623
CHG	20	8	0.621
CHG	20	9	0.618
CHG	20	10	0.615
CHH	1	1	0.603
CHH	2	1	0.602
CHH	2	2	0.604
CHH	3	1	0.603
CHH	3	2	0.604
CHH	3	3	0.604
CHH	4	1	0.603
CHH	4	2	0.604
CHH	4	3	0.604
CHH	4	4	0.604
CHH	5	1	0.603
CHH	5	2	0.604
CHH	5	3	0.604
CHH	5	4	0.604
CHH	5	5	0.603
CHH	6	1	0.603
CHH	6	2	0.604
CHH	6	3	0.604
CHH	6	4	0.604
CHH	6	5	0.603
CHH	6	6	0.603
CHH	7	1	0.603
CHH	7	2	0.604
CHH	7	3	0.604
CHH	7	4	0.604
CHH	7	5	0.603
CHH	7	6	0.603
CHH	7	7	0.603
CHH	8	1	0.604
CHH	8	2	0.604
CHH	8	3	0.604

CHH	8	4	0.604
CHH	8	5	0.603
CHH	8	6	0.603
CHH	8	7	0.603
CHH	8	8	0.602
CHH	9	1	0.602
CHH	9	2	0.603
CHH	9	3	0.603
CHH	9	4	0.603
CHH	9	5	0.602
CHH	9	6	0.602
CHH	9	7	0.602
CHH	9	8	0.602
CHH	9	9	0.602
CHH	10	1	0.603
CHH	10	2	0.603
CHH	10	3	0.603
CHH	10	4	0.603
CHH	10	5	0.602
CHH	10	6	0.602
CHH	10	7	0.602
CHH	10	8	0.602
CHH	10	9	0.602
CHH	10	10	0.603
CHH	11	1	0.603
CHH	11	2	0.603
CHH	11	3	0.603
CHH	11	4	0.603
CHH	11	5	0.602
CHH	11	6	0.602
CHH	11	7	0.602
CHH	11	8	0.602
CHH	11	9	0.602
CHH	11	10	0.603
CHH	12	1	0.603
CHH	12	2	0.603
CHH	12	3	0.603
CHH	12	4	0.603
CHH	12	5	0.602
CHH	12	6	0.602
CHH	12	7	0.602
CHH	12	8	0.602
CHH	12	9	0.602
CHH	12	10	0.603
CHH	13	1	0.603
CHH	13	2	0.603
CHH	13	3	0.602
CHH	13	4	0.602
CHH	13	5	0.602
CHH	13	6	0.602
CHH	13	7	0.601
CHH	13	8	0.601
CHH	13	9	0.601
CHH	13	10	0.603
CHH	14	1	0.603
CHH	14	2	0.603
CHH	14	3	0.602
CHH	14	4	0.602
CHH	14	5	0.602
CHH	14	6	0.602
CHH	14	7	0.601
CHH	14	8	0.601
CHH	14	9	0.601
CHH	14	10	0.603

CHH	15	1	0.602
CHH	15	2	0.602
CHH	15	3	0.602
CHH	15	4	0.602
CHH	15	5	0.601
CHH	15	6	0.601
CHH	15	7	0.601
CHH	15	8	0.601
CHH	15	9	0.601
CHH	15	10	0.603
CHH	16	1	0.602
CHH	16	2	0.602
CHH	16	3	0.601
CHH	16	4	0.601
CHH	16	5	0.601
CHH	16	6	0.601
CHH	16	7	0.601
CHH	16	8	0.601
CHH	16	9	0.601
CHH	16	10	0.603
CHH	17	1	0.602
CHH	17	2	0.602
CHH	17	3	0.601
CHH	17	4	0.601
CHH	17	5	0.601
CHH	17	6	0.601
CHH	17	7	0.601
CHH	17	8	0.601
CHH	17	9	0.601
CHH	17	10	0.603
CHH	18	1	0.602
CHH	18	2	0.602
CHH	18	3	0.601
CHH	18	4	0.601
CHH	18	5	0.601
CHH	18	6	0.601
CHH	18	7	0.601
CHH	18	8	0.601
CHH	18	9	0.601
CHH	18	10	0.603
CHH	19	1	0.602
CHH	19	2	0.602
CHH	19	3	0.601
CHH	19	4	0.601
CHH	19	5	0.601
CHH	19	6	0.601
CHH	19	7	0.601
CHH	19	8	0.601
CHH	19	9	0.601
CHH	19	10	0.603
CHH	20	1	0.601
CHH	20	2	0.601
CHH	20	3	0.601
CHH	20	4	0.601
CHH	20	5	0.600
CHH	20	6	0.601
CHH	20	7	0.600
CHH	20	8	0.600
CHH	20	9	0.600
CHH	20	10	0.602
CpG	1	1	0.556
CpG	2	1	0.556
CpG	2	2	0.555
CpG	3	1	0.556
CpG	3	2	0.555
CpG	3	3	0.546
CpG	4	1	0.556
CpG	4	2	0.555
CpG	4	3	0.546
CpG	4	4	0.546
CpG	5	1	0.556
CpG	5	2	0.554
CpG	5	3	0.546
CpG	5	4	0.546
CpG	5	5	0.534
CpG	6	1	0.555
CpG	6	2	0.554
CpG	6	3	0.546
CpG	6	4	0.546
CpG	6	5	0.534
CpG	6	6	0.532
CpG	7	1	0.555
CpG	7	2	0.554
CpG	7	3	0.546
CpG	7	4	0.546
CpG	7	5	0.534
CpG	7	6	0.532
CpG	7	7	0.507
CpG	8	1	0.554
CpG	8	2	0.554
CpG	8	3	0.546
CpG	8	4	0.546
CpG	8	5	0.534
CpG	8	6	0.532
CpG	8	7	0.506
CpG	8	8	0.508
CpG	9	1	0.555
CpG	9	2	0.554
CpG	9	3	0.545
CpG	9	4	0.546
CpG	9	5	0.534
CpG	9	6	0.532
CpG	9	7	0.506
CpG	9	8	0.508
CpG	9	9	0.502
CpG	10	1	0.554
CpG	10	2	0.553
CpG	10	3	0.545
CpG	10	4	0.546
CpG	10	5	0.534
CpG	10	6	0.532
CpG	10	7	0.506
CpG	10	8	0.508
CpG	10	9	0.502
CpG	10	10	0.506
CpG	11	1	0.553
CpG	11	2	0.552
CpG	11	3	0.545
CpG	11	4	0.546
CpG	11	5	0.534
CpG	11	6	0.532
CpG	11	7	0.506
CpG	11	8	0.508
CpG	11	9	0.502
CpG	11	10	0.506
CpG	12	1	0.552
CpG	12	2	0.551
CpG	12	3	0.545

CpG	12	4	0.546
CpG	12	5	0.534
CpG	12	6	0.532
CpG	12	7	0.506
CpG	12	8	0.508
CpG	12	9	0.502
CpG	12	10	0.506
CpG	13	1	0.554
CpG	13	2	0.552
CpG	13	3	0.547
CpG	13	4	0.548
CpG	13	5	0.536
CpG	13	6	0.534
CpG	13	7	0.508
CpG	13	8	0.509
CpG	13	9	0.501
CpG	13	10	0.505
CpG	14	1	0.553
CpG	14	2	0.552
CpG	14	3	0.547
CpG	14	4	0.548
CpG	14	5	0.536
CpG	14	6	0.534
CpG	14	7	0.508
CpG	14	8	0.509
CpG	14	9	0.501
CpG	14	10	0.505
CpG	15	1	0.554
CpG	15	2	0.552
CpG	15	3	0.548
CpG	15	4	0.548
CpG	15	5	0.536
CpG	15	6	0.534
CpG	15	7	0.508
CpG	15	8	0.509
CpG	15	9	0.501
CpG	15	10	0.505
CpG	16	1	0.553
CpG	16	2	0.552
CpG	16	3	0.548
CpG	16	4	0.548
CpG	16	5	0.536
CpG	16	6	0.534
CpG	16	7	0.508
CpG	16	8	0.509
CpG	16	9	0.501
CpG	16	10	0.505
CpG	17	1	0.551
CpG	17	2	0.551
CpG	17	3	0.547
CpG	17	4	0.548
CpG	17	5	0.536
CpG	17	6	0.534
CpG	17	7	0.508
CpG	17	8	0.509
CpG	17	9	0.501
CpG	17	10	0.505
CpG	18	1	0.552
CpG	18	2	0.551
CpG	18	3	0.548
CpG	18	4	0.549
CpG	18	5	0.536
CpG	18	6	0.534
CpG	18	7	0.508
CpG	18	8	0.509
CpG	18	9	0.501
CpG	18	10	0.505
CpG	19	1	0.551
CpG	19	2	0.550
CpG	19	3	0.549
CpG	19	4	0.549
CpG	19	5	0.536
CpG	19	6	0.534
CpG	19	7	0.508
CpG	19	8	0.509
CpG	19	9	0.501
CpG	19	10	0.505
CpG	20	1	0.550
CpG	20	2	0.550
CpG	20	3	0.549
CpG	20	4	0.550
CpG	20	5	0.537
CpG	20	6	0.535
CpG	20	7	0.509
CpG	20	8	0.510
CpG	20	9	0.501
CpG	20	10	0.504
